# GTRD: an integrated view of transcription regulation

**DOI:** 10.1093/nar/gkaa1057

**Published:** 2020-11-24

**Authors:** Semyon Kolmykov, Ivan Yevshin, Mikhail Kulyashov, Ruslan Sharipov, Yury Kondrakhin, Vsevolod J Makeev, Ivan V Kulakovskiy, Alexander Kel, Fedor Kolpakov

**Affiliations:** BIOSOFT.RU, LLC, Novosibirsk 630090, Russian Federation; Federal Research Center for Information and Computational Technologies, Novosibirsk 630090, Russian Federation; Federal Research Center Institute of Cytology and Genetics SB RAS, Novosibirsk 630090, Russian Federation; BIOSOFT.RU, LLC, Novosibirsk 630090, Russian Federation; Federal Research Center for Information and Computational Technologies, Novosibirsk 630090, Russian Federation; BIOSOFT.RU, LLC, Novosibirsk 630090, Russian Federation; Federal Research Center for Information and Computational Technologies, Novosibirsk 630090, Russian Federation; Novosibirsk State University, Novosibirsk 630090, Russian Federation; BIOSOFT.RU, LLC, Novosibirsk 630090, Russian Federation; Federal Research Center for Information and Computational Technologies, Novosibirsk 630090, Russian Federation; Novosibirsk State University, Novosibirsk 630090, Russian Federation; BIOSOFT.RU, LLC, Novosibirsk 630090, Russian Federation; Federal Research Center for Information and Computational Technologies, Novosibirsk 630090, Russian Federation; Vavilov Institute of General Genetics RAS, Moscow 119991, Russian Federation; Moscow Institute of Physics and Technology (State University), Dolgoprudny 141700, Russian Federation; NRC «Kurchatov Institute» - GOSNIIGENETIKA, Kurchatov Genomic Center, Moscow 123182, Russian Federation; Engelhardt Institute of Molecular Biology, Russian Academy of Sciences, Moscow 119991, Russian Federation; Vavilov Institute of General Genetics RAS, Moscow 119991, Russian Federation; Engelhardt Institute of Molecular Biology, Russian Academy of Sciences, Moscow 119991, Russian Federation; Institute of Protein Research, Russian Academy of Sciences, Pushchino 142290, Russian Federation; BIOSOFT.RU, LLC, Novosibirsk 630090, Russian Federation; geneXplain GmbH, 38302 Wolfenbüttel, Germany; Institute of Chemical Biology and Fundamental Medicine SB RAS, Novosibirsk 630090, Russian Federation; BIOSOFT.RU, LLC, Novosibirsk 630090, Russian Federation; Federal Research Center for Information and Computational Technologies, Novosibirsk 630090, Russian Federation

## Abstract

The Gene Transcription Regulation Database (GTRD; http://gtrd.biouml.org/) contains uniformly annotated and processed NGS data related to gene transcription regulation: ChIP-seq, ChIP-exo, DNase-seq, MNase-seq, ATAC-seq and RNA-seq. With the latest release, the database has reached a new level of data integration. All cell types (cell lines and tissues) presented in the GTRD were arranged into a dictionary and linked with different ontologies (BRENDA, Cell Ontology, Uberon, Cellosaurus and Experimental Factor Ontology) and with related experiments in specialized databases on transcription regulation (FANTOM5, ENCODE and GTEx). The updated version of the GTRD provides an integrated view of transcription regulation through a dedicated web interface with advanced browsing and search capabilities, an integrated genome browser, and table reports by cell types, transcription factors, and genes of interest.

## INTRODUCTION

Transcriptional regulation is a complex process that depends on multiple factors (1,2), including:

- transcription factors (TFs), which bind the DNA sites in the gene regulatory regions and activate or repress gene expression through interaction with various cofactors;
- histone modifications (methylation, phosphorylation, acetylation, etc.) that define the state of chromatin and make particular regions active or inactive;
- DNA methylation of cytosine or adenine, which changes the activity of individual promoters or even large loci, mainly by suppressing gene expression and promoting the formation of heterochromatin.

High-throughput sequencing provides a way to assess those factors at the genome-wide scale. Large-scale international collaborations such as ENCODE (3) and FANTOM (4) serve as the gold standard for systematic acquisition, integrative analysis, and sharing of high-quality experimental data. In particular, the ENCODE Encyclopedia brings together the most salient analytical products and provides tools for searching and visualizing these data (5). However, despite being the largest single source, the ENCODE data provide only a limited contribution to the total pool of the data on gene regulation produced in different research laboratories. Such data are available in GEO (6) and SRA (7) repositories but lack uniform annotation and processing pipelines, thus limiting the possibilities for large-scale integrated analysis. This motivates the development and application of standardized workflows and databases to allow for efficient usage of the vast but unsystematic published data.

In pursuing this goal, we started developing the Gene Transcription Regulation Database (GTRD) in 2011. We started with uniform annotation and analysis of key components of transcription regulation – transcription factor binding sites (TFBSs) – identified in ChIP-seq experiments for humans and mice, the top two species by the number of ChIP-seq experiments in GEO (8). In the next major release, we extended the GTRD content by seven additional species using the most available TF ChIP-Seq experiments (the complete list: *Homo sapiens, Mus musculus, Rattus norvegicus, Danio rerio, Caenorhabditis elegans, Drosophila melanogaster, Saccharomyces cerevisiae, Schizosaccharomyces pombe* and *Arabidopsis thaliana*), and included the data on the next major level of transcription regulation – the open chromatin regions identified in DNase-seq experiments (9).

The current goal of the GTRD database is to provide uniform annotation and integrative analyses of all next-generation sequencing (NGS) data from GEO and SRA that meet the following criteria: (1) related to transcription regulation; (2) already widespread and actively generated by the research community. With these ideas in mind, we included the following experimental data types:

- ChIP-exo (10) and ChIP-nexus (11) for precise identification of TFBSs,
- ChIP-seq (12) for identification of the histone modifications,
- ATAC-seq (13) and FAIRE-seq (14) for identification of open chromatin regions,
- MNase-seq (15) for assessing nucleosome landscapes,
- RNA-seq from cells with altered TF activity through (knock-out, knock-down), or various types of activation to reveal the TF target genes (16).

**Figure 1.**
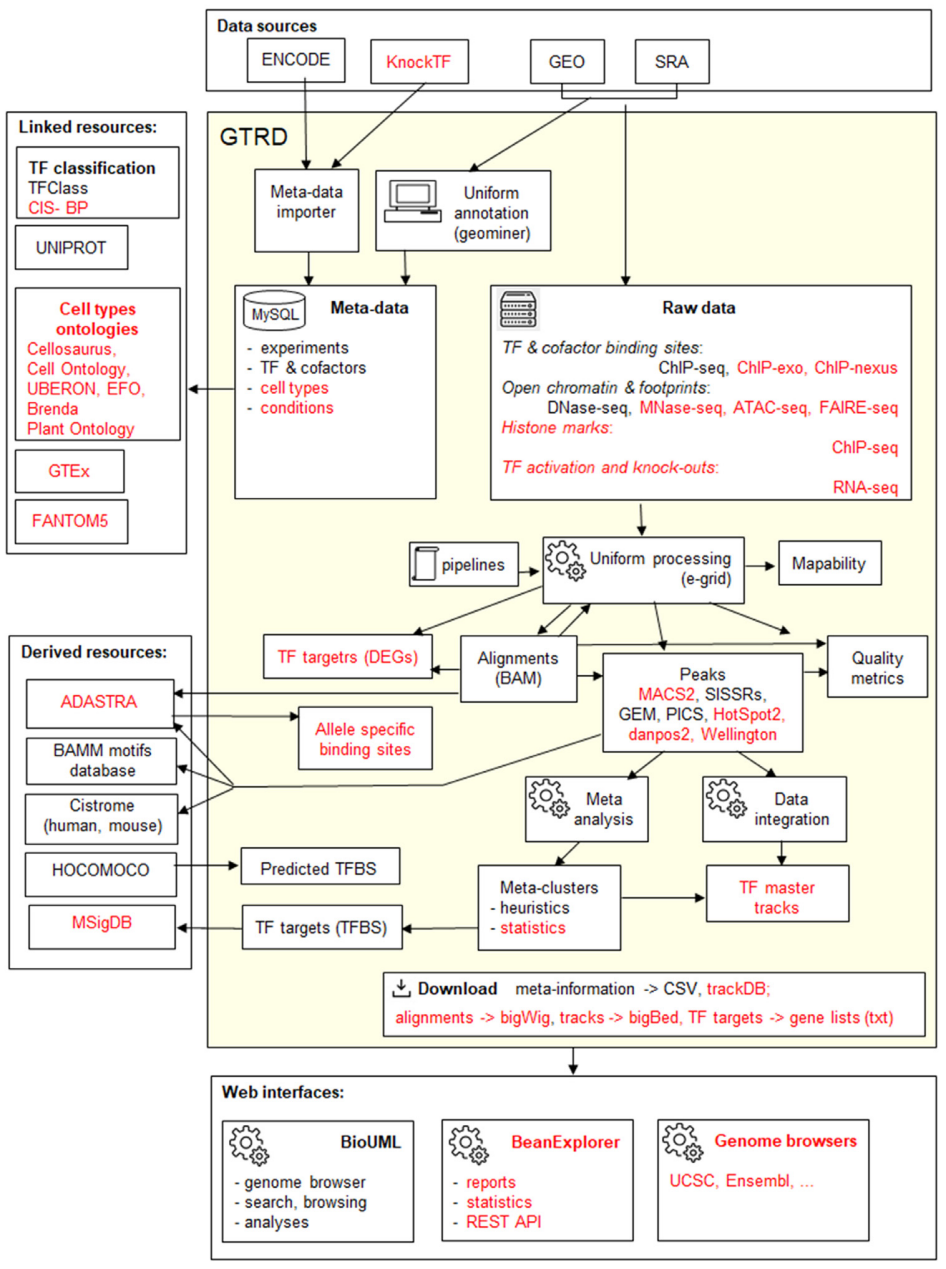
Current workflow for uniform annotation and analysis of experimental data in the GTRD. New data and tools added in the most recent version compared to the previous publication are highlighted in red. DEGs – differentially expressed genes.

We also provide results of extended analysis of the previously included data, such as updated meta-clusters (8), master-TFBS tracks and allele-specific binding sites derived from TF ChIP-Seq data. Below, we briefly describe the current workflow allowing for uniform annotation and analysis of the experimental data in the GTRD database as well as integration with other resources related to transcription regulation (Figure 1), highlighting changes in the current release relative to previous GTRD publications (8,9).

## MATERIALS AND METHODS

GTRD content is divided into four sections:

1. Raw data downloaded from GEO, SRA, ENCODE and modENCODE.
2. Meta-data generated by automated annotation and expert curation of the raw data. The meta-data contains the uniform annotation of experiments (cell source, experimental conditions, control experiment) and information specific to the data type (e.g. for ChIP-seq experiments, the target TF, and the used antibody). We have developed automation tools that download meta-information from ENCODE, modENCODE, and KnockTF (16) and reduce the efforts for manual annotation. Particularly, a special geominer software (Supplement 1) is used for proper conversion and annotation of the original GEO metadata with control vocabularies and ontologies.
3. Results of uniform analysis of the raw data. For each type of experiment, we developed specialized workflows for uniform analysis (http://wiki.biouml.org/index.php/GTRD_Workflow). These workflows are executed within our in-house distributed computing solution, e-grid, which allows parallel data processing on multiple computational nodes.
4. Downloads, the plain text files that contain results of the uniform annotation and analysis of the data described above, which can be used for automated downstream analysis of gene regulation by external tools.

Previous versions of the GTRD used the MySQL database as a backend to store the meta-data and all generated tracks (except for the read alignment BAM files). As the data volume increased, the backend became a bottleneck, and in the recent release, the MySQL database is used only for meta-data. The generated genomic signal tracks are stored directly in the file system as bigBed (17) files, providing multiple advantages:

- bigBed processing is significantly more efficient in e-grid in parallel mode;
- the storage space requirements are much lower due to bigBed internal compression;
- bigBed files can be directly downloaded from the GTRD without additional conversion and directly visualized in UCSC (17) or Ensembl (18) genome browsers.

The alignment data is also available for download in bigWig format (17) suitable for visualization in external genome browsers. Particularly, bigWig files are provided for ChIP-seq peaks coverage, DNase-seq and ATAC-seq coverage of open chromatin regions, and gene expression estimates from RNA-seq data.

We have updated the links to the classification of human and mouse TFs with the most recent TFClass data (19), which allows the use of the GTRD with the database of transcription factors and their motifs, TRANSFAC (20). Furthermore, in this GTRD release, we also linked the TFs with CIS-BP (21), which covers all model organisms of GTRD (except *Schizosaccharomyces pombe*).

An essential task for integrative analysis is the accurate annotation of the experimental data, including experimental conditions with suitable control vocabularies and ontologies. In the current version of the GTRD, we created a single dictionary for cell types that includes 3954 entries (22). For further analyses and visualization, the cell types were divided into 90 clusters corresponding to the main organs and tissues of corresponding organisms. Whenever possible, all entries were linked with the existing cell type dictionaries and ontologies: UBERON (23), Cell Ontology (24), BRENDA tissue ontology (25), Plant Ontology (26), and Cellosaurus (27). A dictionary of experimental conditions was also created and linked with the Experimental Factor Ontology (EFO) (28). Links to these ontologies allow accurate mapping of GTRD data with data from other databases on gene expression regulation, taking into account cell specificity and experimental conditions. This way (8), the GTRD data was arranged with the relevant data on gene expression from FANTOM5 (29) and GTEx (30).

We have developed new methods of integrative analysis, as described below.

### Construction of meta-clusters with statistical methods

The meta-clusters are sets of non-redundant TFBSs for each TF that are obtained by merging TFBSs identified by different peak callers in different experiments for the same TF. Previously, we used a heuristic approach (8). Here, we applied a novel algorithm based on quantitative quality metrics (31) and rank aggregation (32) (see Supplement 2 for algorithm details). This not only allows the generation of new versions of meta-clusters but also provides reliability measures in the form of rank aggregation scores, allowing the most reliable meta-clusters to be picked up. Two approaches are suitable for selection of the most reliable meta-clusters (Supplement 3). The first approach is based on a two-component normal mixture, the second uses logistic regression. In general, the most reliable meta-clusters are in good concordance with meta-clusters found with the previous heuristic version (Supplement 4), but have shorter lengths. However, truly reliable meta-clusters require taking into account not only statistical scores but also biological data (location within open chromatin regions, DNase footprints, sequence motifs, etc). To include this information, we introduced the concept of the master track.

### Construction of master tracks

The track of the master sites is a further extension of the concept of meta-clusters. For a given TF, the meta-cluster defines the boundaries of the binding site (binding region). The master site for such a region integrates all information from the GTRD: set of peaks used for meta-cluster construction (ChIP-seq, ChIP-exo); motif hits from sequence scanning with position weight matrices (PWMs); cell types where corresponding peaks were identified; overlapping open chromatin regions and DNase or ATAC-seq footprints; allele-specific binding events. This information provides important data on TF binding in different cell types and conditions. The BioUML genome browser provides visualization of this information (Supplement 5).

**Identification of the allele-specific binding (ASB) events** occurs where a TF demonstrates differential binding to alternating alleles of a single-nucleotide variant (33).

Another important practical task is the identification of TF target genes. A common approach is to consider a gene to be regulated by a given TF if it has a corresponding binding site in the promoter region. For all TFs in the GTRD, we generated lists of genes with binding sites in the promoter region, which is defined in three variants—[−5000; +500], [–1000; +100], [–500; +50] nt relative to the transcription start sites (TSSs)—and in the whole gene [−5000, +5000] relative to gene boundaries. These lists are also included in MSigDB (34) for gene set enrichment analysis. It is noteworthy that only a limited fraction of TFBSs (typically <15%) directly affects gene expression; thus, while being useful for exploratory analysis, these data should be used with caution in other scenarios, such as reconstruction of regulatory networks.

The new GTRD version also provides TF targets revealed by another common approach: analysis of RNA-seq data from cells with down- or upregulated TF activity (knock-out, knock-down, or activation experiments). Depending on experiment conditions, such data can still reveal indirect TF targets—e.g., if RNA-seq is performed later than one hour after corresponding TF activation.

The new GTRD release features a significantly updated web interface provided by the BioUML platform (9): it provides a data browser, advanced search capabilities, and an integrated genome browser for all data types included in the GTRD. A novel ‘Track finder’ panel was added to the BioUML genome browser for advanced search of genomic tracks by different criteria (see Supplement 6).

**Figure 2.**
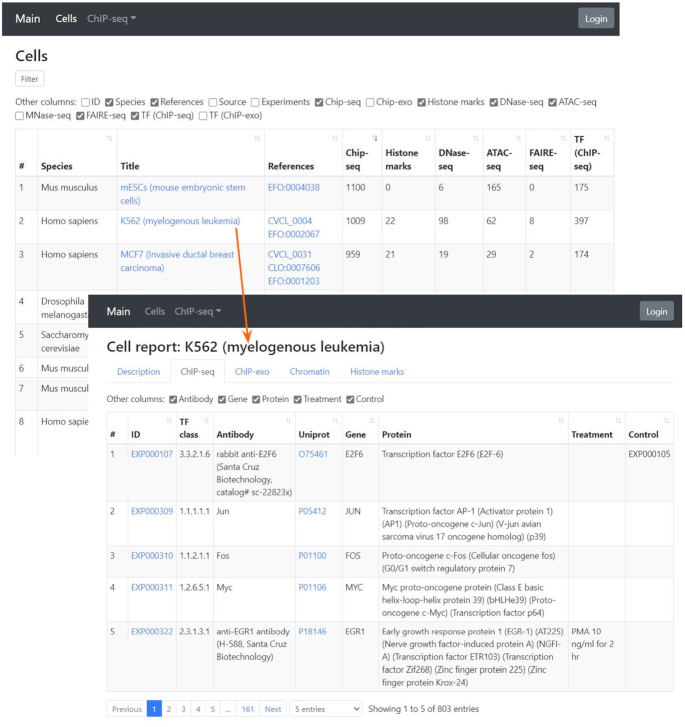
Example of a summary report and an overview of data for a selected cell type.

We also present a completely new web interface for generating summary statistics and reports for a given cell type, TF, and experiment from GTRD meta-data (Figure 2). Here, we applied the BeanExplorer technology (https://github.com/DevelopmentOnTheEdge/beanexplorer) that easily allows to generate a web interface based on information from any relational database.

**Figure 3.**
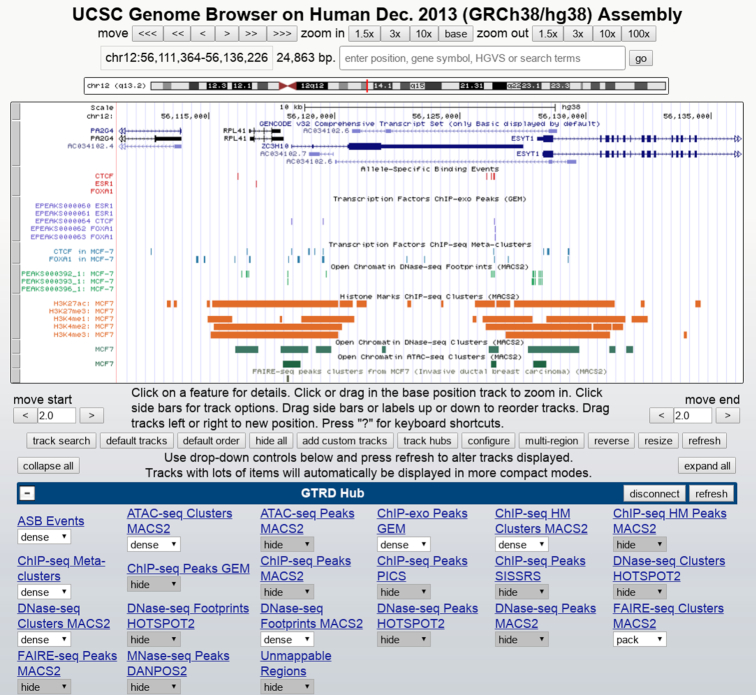
Visualization of GTRD tracks in the UCSC genome browser.

The bigBed and Big files from the GTRD website can be directly visualized by the UCSC (36) and Ensembl (18) genome browsers (Figure 3) with the dedicated track hubs created for each species. Each track hub contains main tracks from the GTRD database in bigBed and bigWig formats and the necessary meta-information following the UCSC track hub standards (37).

We would like to highlight that information from the GTRD was successfully used in several derived resources, such as: (i) the HOCOMOCO collection of TFBS models (38), which is based on GTRD ChIP-Seq peaks and provides the resulting models for usage within the GTRD for genome scanning and TFBS recognition within ChIP-Seq peaks; (ii) the BaMM motifs database (39) and the BaMM server (40) for the recognition of TFBSs; (iii) human and mouse cistromes, the maps of putative cis-regulatory regions bound by TFs, as an early approach to the construction of meta-clusters (41); (iv) MSigDB (34) includes a GTRD subset of TF targets—genes contain TFBSs identified in ChIP-seq experiments in the region [−1000,+500] nt around the TSS; (v) ADASTRA database of allele-specific binding sites (https://adastra.autosome.ru/).

## DISCUSSION

TF ChIP-seq was the first type of experimental data reprocessed and provided in the GTRD, which remains the largest database by the number of TFs for which available NGS data (ChIP-seq, ChIP-exo, ChIP-nexus) were uniformly annotated and processed (Table 1). The number of ChIP-seq experiments in the GTRD has doubled since our previous publication (35 719 reported in the current release versus 17 485 in the previous one). Importantly, the GTRD is continuously updated, and all ChIP-seq experiments from GEO and SRA for nine taxa within the GTRD scope of interest are usually included within six months after being deposited in public access.

**Table 1.** Comparison of the GTRD with other databases on ChIP-seq experiments

Database	Number of TF ChIP-seq experiments	Number of TFs	ChIP-seq peak callers	Species
*GTRD v20.06*	total: 35 719 *H. sapiens:* 15 982	total: 3 599 *H. sapiens:* 1391	MACS2, MACS, GEM, PICS, SISSRs	*H. sapiens, M. musculus, R. norvegicus,D. melanogaster, C. elegans, D. rerio, S. pombe, S. cerevisiae, A. thaliana*
ChIP-Atlas	total: 30 495*H. sapiens: 13 558*	total: 1 781**H. sapiens: 1 020**	MACS2	*H. sapiens, M. musculus, R. norvegicus, D. melanogaster, C. elegans, S. cerevisiae*
CistromeDB	total: 24 065**H. sapiens: 13 976**	total: 1654**H. sapiens: 1470**	MACS2	*H. sapiens, M. musculus*
ENCODE	total: 3 816 *H. sapiens:* 2 632	total: 2 160 *H. sapiens:* 964	SPP	*H. sapiens, M. musculus, D. melanogaster, C. elegans*
ChIPBase	total: 4300**H. sapiens: 2498**	total: 870**H. sapiens: 480**	no uniform pipeline, each ChIP-seq is processed by different peak caller	*H. sapiens, M. musculus, R. norvegicus,D. rerio, X. tropicalis, C. elegans,D. melanogaster, S. cerevisiae, A. thaliana, G. gallus*
ReMap 2020 3rd release	total: 6307**H. sapiens: 5798**	total: 1507**H. sapiens: 1135**	MACS2	*H. sapiens, A. thaliana*
Factorbook	total: 1886**H. sapiens: 1813**	total: 682**H. sapiens: 682**	None	*H. sapiens, M. musculus*

*‘TF and others’ according to ChIP-Atlas info. 7 374 input files for human and 17 914 input files in total given in a separate category in ChIP-Atlas are not taken into account in the table as they are not assigned to particular ChIP-Seq data.

**TFs and other DNA binding proteins excluding polymerases and histones.

There is a practically important question: What is the total fraction of known TFs covered by ChIP-seq, ChIP-exo, or ChIP-nexus experiments? For this purpose, we linked TFs in the GTRD with CIS-BP, which contains the most comprehensive TF lists across species (Table 2). Notably, about two-thirds of human TFs are covered by at least one ChIP-Seq experiment, so in a few years, we can expect that genomic binding-site data will be available for all human TFs experimentally studied in at least one cell type. This holds also for *D. melanogaster* and *S. cerevisiae*. A special section in the GTRD is under construction to highlight TFs without published ChIP-seq, ChIP-exo, or ChIP-nexus experiments, so scientists can share their research status for a given TF – whether it is under investigation now or planned for the near future, and when results will be publicly available.

**Table 2.** Coverage of known TFs by ChIP-seq, ChIP-exo, and ChIP-nexus experiments in the GTRD database

Specie	CIS-BP (TF)	GTRD (TF & cofactors)	TF - intersecton CIS-BP - GTRD	%
*Homo sapiens*	1639	1535	1032	63
*Mus musculus*	1513	856	473	31
*Rattus norvegicus*	1362	47	19	1.4
*Danio rerio*	2350	27	13	0.5
*Caenorhabditis elegans*	766	338	218	28.5
*Drosophila melanogaster*	719	583	360	50
*Saccharomyces cerevisiae*	239	198	-	21
*Schizosaccharomyces pombe*	115	66	-	8.6
*Arabidopsis thaliana*	1749	135	88	5

**Figure 4.**
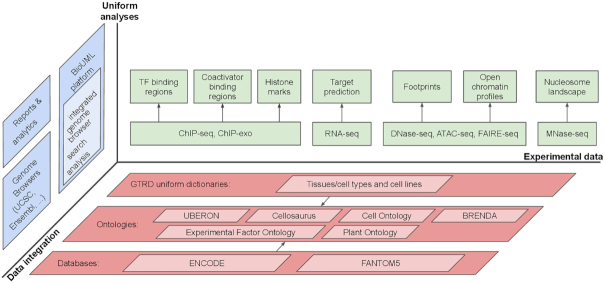
GTRD – integrated view of transcription regulation.

The current version of the GTRD is not limited to TF ChIP-seq data, and the contribution of other data is increasing (see summary statistics in Supplements 7, 8). Figure 4 illustrates our vision of gene transcription regulation data and its integrative analysis and representation within the GTRD framework. We also plan to integrate data on methylation of DNA into the GTRD. The MethMotif database (42) demonstrates how ChIP-seq data can be integrated with cell type-specific CpG methylation information.

Integration of the GTRD with other collections of experimental data on transcription regulation (FANTOM5, ENCODE, and GTEx) can act as a starting point for studying specific questions of mechanisms of transcription regulation of specific genes. For example, in our study of connections between TSS activities and the TFBSs (Sharipov *et al.*, 2020, accepted in *PLoS One*), we developed an algorithm that predicts gene expression from TFBSs using FANTOM5 gene expression data and TF binding data from the GTRD. The algorithm utilizes precise TFBS locations and their arrangements, correlating them with TSS activity identified in the FANTOM5 project, thus yielding TFs contributing most to the control of gene expression and the location of their binding sites as referred to TSS.

Single-nucleotide variants (SNV) in gene regulatory regions can alter gene expression and contribute to phenotypes of individual cells and the whole organism, including disease susceptibility and progression. The Genotype-Tissue Expression (GTEx) project provides information about associations of SNV and gene expression for 54 non-diseased tissues (eQTL). Using clusters of cell types described above, we can associate eQTL data with corresponding TFBSs, taking into account the cellular context to study possible mechanisms of SNVs’ alternations of gene activity.

Thus, with this latest release, the GTRD database becomes the largest integrated resource of data on transcription regulation in eukaryotes. It is noteworthy that GTRD contains not only uniformly annotated and processed NGS data, but also the results of the meta-analysis, presented in the form of meta-clusters, the sets of non-redundant and reproducible TFBSs for each TF, obtained by merging TFBSs identified in different experiments for the same TF. The meta-clusters can be considered as the first step towards complete cistrome for the corresponding organisms. The track of the master sites that integrates all relevant information from the GTRD database for a given TFBS further extends the concept of meta-clusters. This information can be used both for understanding how a particular site is involved in the transcription regulation of a gene as well as and for the development of new methods for identification of the most reliable TFBS from both statistical and biological evidence.

## Supplementary Material

gkaa1057_Supplemental_File
