# Supplementary material for: GTRD: an integrated view of transcription regulation
Source: Nucleic Acids Res. 2020 Nov 24;49(D1):D104–11. doi: 10.1093/nar/gkaa1057 (PMC7778956; doi:10.1093/nar/gkaa1057)
Supplement: gkaa1057_Supplemental_File [file gkaa1057_supplemental_file.docx]

## SUPPLEMENTARY DATA

**Supplement 1**

**Geominer tool**

Figure S1 demonstrates the interface of our tool, Geominer, which helps annotators to add information for GTRD experiments.

Panel 1 – describes the information about GSE and contains some external links – for example, to GSE series and to publications in PubMed, if work was published.

Panel 2 – presents parsed information added to GSE by authors for every GSM.

Panel 3 – is used to choose GSMs by annotator from the drop-down list. Also in panel 3, the annotator can merge some GSMs if there is insufficient control for them. The “Remove” button is used to remove some GSMs from the annotation, for reasons such as there being insufficient data or if the experiment type is not presented in the GTRD. The “Reject” button is used to remove GSE from the annotation for the same reasons described above for “Remove.”

Panel 4 – is used by the annotator to add information in specialized fields, which will be added to GTRD databases for chosen GSMs.


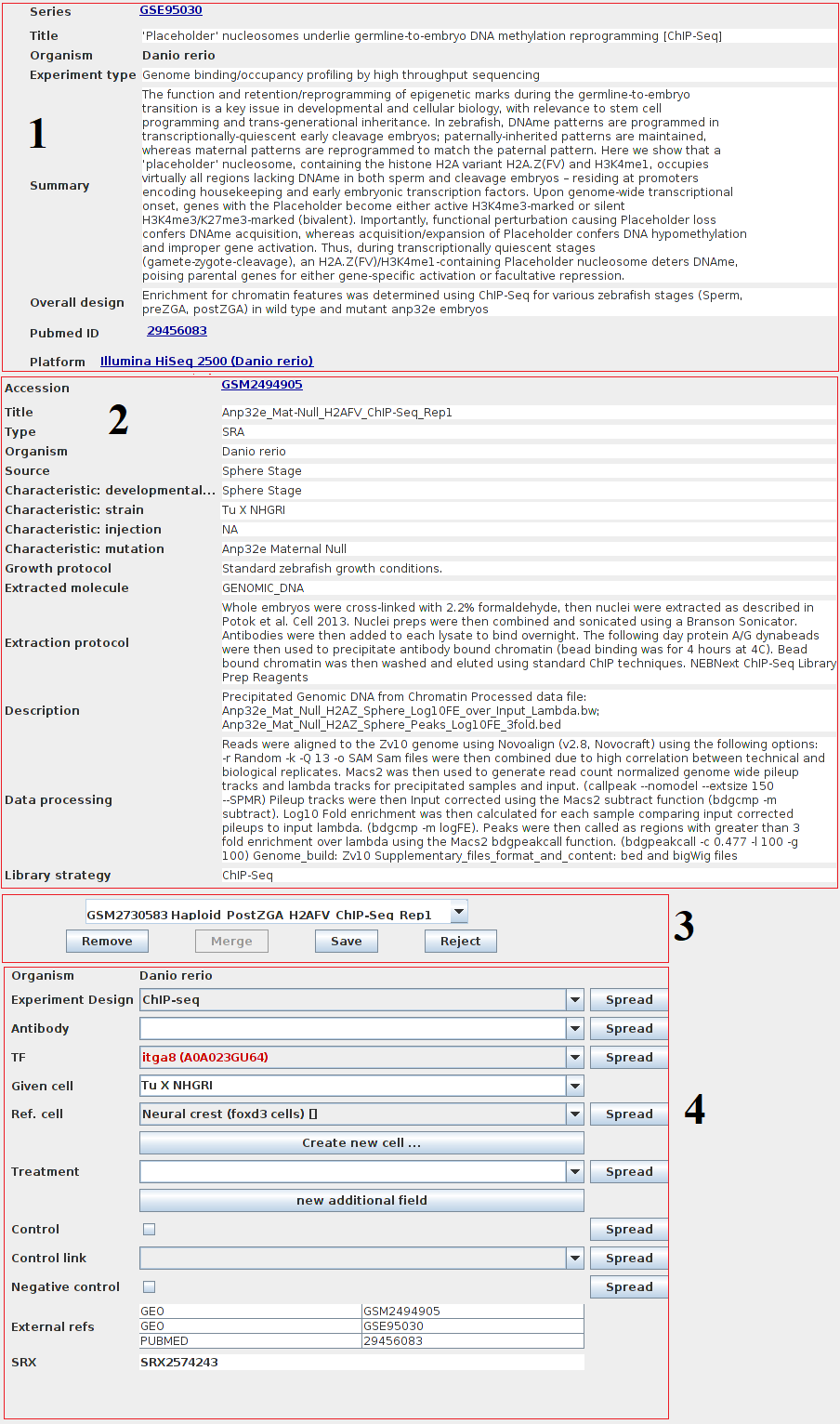


**Figure S1**. Geominer interface.

**Supplement 2**

Novel algorithm for meta-cluster construction

For the generation of new versions of meta-clusters, we developed a novel algorithm based on RA (Rank Aggregation) and the quality control approach [1] of the initial ChIP-seq data. In general, the RA approach is widely used to perform meta-analysis of different types of data. It is successively applied in many fields of knowledge such as political elections, marketing, and document analysis. In bioinformatics, the RA approach has usually been used to obtain the final list of genes (e.g., associated with a disease or traits) from several lists of genes identified in different studies [2, 3, 4]. However, the RA approach has not been used for meta-analyses of ChIP-seq data.

Our algorithm consists of three steps of meta-processing of ChIP-seq data. It generates not only new versions of meta-clusters but also RA-scores assigned to each meta-cluster. These RA-scores allow analysis and selection of the most reliable meta-clusters. As the first step of the algorithm, we applied, independently, four peak callers (GEM, MACS2, PICS, and SISSRs) to the same data obtained from an individual ChIP-seq experiment. As a rule, peak caller assigns several quality characteristics (such as “Fold enrichment” and “FDR”) to each generated peak. On the basis of these characteristics, we generated a single RA1-score for each peak (see Figure S2). For this purpose, we used the simple arithmetic mean.

As the second step of the algorithm, all available peaks were overlapped and quality control was performed. For this purpose, we used the FPCM (False Positive Control Metric) [1]. This metric controls the falsely generated “orphans” – i.e., peaks that do not overlap with other peaks. If the FPCM exceeded a threshold of 3.0, all orphans were discarded, because the majority of them were classified as falsely generated [1]. As a result, new RA2-scores were computed for overlapped peaks as the arithmetic means of ranks of RA1-scores. Thus, the single dataset of overlapped peaks with RA2-scores was identified for the given ChIP-Seq experiment for a given transcription factor.

As the third step, the single set of meta-clusters for a given TF was obtained by overlapping all datasets of overlapped peaks obtained in the second stage for that given TF. Final RA3-scores were evaluated for the obtained meta-clusters, also as the arithmetic means of ranks of RA2-scores.


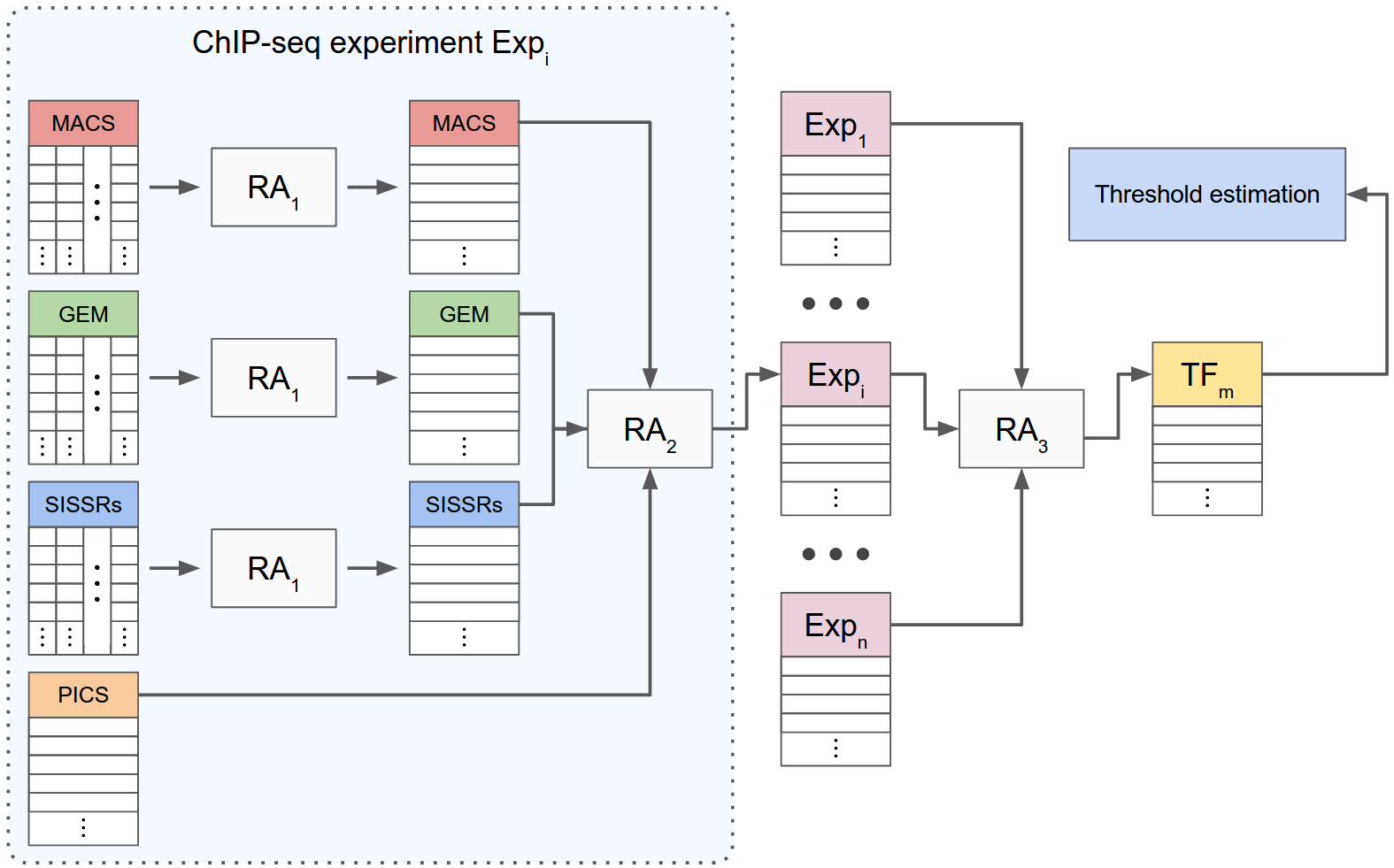


**Figure S2.** Workflow of the algorithm for meta-cluster construction. RAn – n-th rank aggregation stage.

**Supplement 3**

Two ways for selection of the most reliable meta-clusters

In general, the RA approach only allows sorting of the analyzed meta-clusters in order of increasing reliability. But this approach did not recommend how to define the threshold for choosing the most reliable meta-clusters. We have considered two ways for selection of the most reliable meta-clusters. The first approach is based on a two-component normal mixture, while the second is based on the construction of classification models of the logistic regression type.

To determine the RA-score threshold, we used an algorithm for estimation of the two components of the normal mixture. In other words, we suggested that the total set of RA-scores consisted of RA-scores of truly identified meta-clusters and RA-scores of falsely identified meta-clusters. Thus, it was suggested that the total set of RA-scores is a mixture of two normal distributions N1(α1, σ12) and N2(α2, σ22); the unknown parameters α1, σ12, α2, and σ22 were estimated by the Expectation-Maximization Algorithm. Obviously, when α1< α2, the distribution N1(α1, σ12) responds to RA-scores of the truly identified meta-clusters, while N2(α2, σ22) responds to the RA-scores of the falsely identified meta-clusters. We defined the RA-score threshold as T = α2 − 1.645 * σ2. According to the definition of the standard normal distribution, such determination of T ensures that the majority (95%) of falsely identified meta-clusters (for which RA-scores > T) are classified as less reliable meta-clusters. The meta-clusters for which RA-scores < T are classified as the most reliable meta-clusters.

The keystone of the second approach is the construction of a classification model of the logistic regression type. For this purpose, we selected two subsets of 20,000 meta-clusters with the highest and lowest RA-scores and applied the logistic regression to them. To discriminate these lists, we used the following five features: RA-scores, squared RA-scores, motif scores, squared motif scores, and the interactions between RA-scores and site motifs, which were expressed as products of RA-scores and motif scores. For motif identification, we used a position weight matrix (PWM) approach – namely, HOCOMOCO PWMs. Then, we used the constructed classification model to assign probability Pr to every meta-cluster in the total set. In this case, the Pr was defined as the probability that a given meta-cluster can be the most reliable meta-cluster. Hence, the most reliable subset of meta-clusters can be composed by selection of meta-clusters for which Pr exceeds the pre-specified threshold Pr0 – for example, Pr0 = 0.95 or 0.99. It is important to note that the normal mixture algorithm was able to identify two normal components for almost all sets of meta-clusters. However, this was not the case for classification models, due to two reasons. First, for many TFs, the corresponding HOCOMOCO PWMs were not available. Second, the accuracy of many constructed classification models was unsatisfactory.

**Supplement 4**

Comparative analysis of old and new meta-clusters

To compare new and old meta-clusters, we compared their lengths and numbers. In general, the total number of new meta-clusters increased. However, the number of new most reliable meta-clusters is comparable with the number of old meta-clusters. In particular, Table S1 contains information about lengths and numbers of meta-clusters for ESR1, JUN, and FOXA1. Regarding the lengths, there was a strong tendency for the majority of TFs: on average, the new most reliable meta-clusters are shorter than old meta-clusters, while new less reliable meta-clusters are longer then old meta-clusters (see Table S1). Additionally, Figure S3 contains densities of meta-cluster lengths for ESR1, JUN, and FOXA1.

Finally, we also compared the site motif abundance in meta-clusters. To analyze site motifs within meta-clusters, we built ROC (receiver operating characteristic) curves and calculated the AUCs (areas under curves) for each set of meta-clusters for ESR1, JUN, and FOXA1. For motif identification, we used the PWM approach and the following HOCOMOCO PWMs: ESR1_HUMAN.H11MO.0.A, JUN_HUMAN.H11MO.0.A, and FOXA1_HUMAN.H 11 MO.0.A. Figure S4 and Table S2 contain the ROC curves and the corresponding AUCs. According to AUC values, one can conclude that the most reliable meta-clusters were highly saturated with site motifs for ESR1 and FOXA1, while old meta-clusters were highly saturated with site motifs for JUN. Finally, it is interesting to note that if we consider only the top lists of the 20,000 most reliable meta-clusters, then AUC values are essentially increased. Thus, AUCs achieved the following values: 0.806 (for ESR1), 0.729 (for JUN), and 0.945 (for FOXA1). Additionally, we also calculated the AUC values for the same three TFs, with the help of TRANSFAC PWMs [5] (see Table S2). For this purpose, we used V$ESR1_10, V$JUN_11, and V$FOXA1_14 matrices from the TRANSFAC database [6]. The transition from HOCOMOCO PWMs to TRANSFAC PWMs confirmed the conclusion that the most reliable meta-clusters were highly saturated with site motifs in comparison with the less reliable meta-clusters (see Table S2). In other words, this conclusion was invariant with respect to the choice of PWMs.

**Table S1.** Summary on meta-clusters for ESR1, JUN, and FOXA1.

| **TF** | **Type of meta-clusters** | **Number of meta-clusters** | **Mean length of meta-clusters** |
| --- | --- | --- | --- |
| ESR1 | Old meta-clusters | 3 840 967 | 85.245 |
|  | New most reliable meta-clusters | 1 322 746 | 35.346 |
|  | New less reliable meta-clusters | 6 281 934 | 97.939 |
| JUN | Old meta-clusters | 651 010 | 71.881 |
|  | New most reliable meta-clusters | 726 591 | 44.845 |
|  | New less reliable meta-clusters | 589 169 | 155.963 |
| FOXA1 | Old meta-clusters | 1 388 959 | 69.054 |
|  | New most reliable meta-clusters | 1 037 794 | 34.625 |
|  | New less reliable meta-clusters | 1 666 722 | 135.311 |

a)
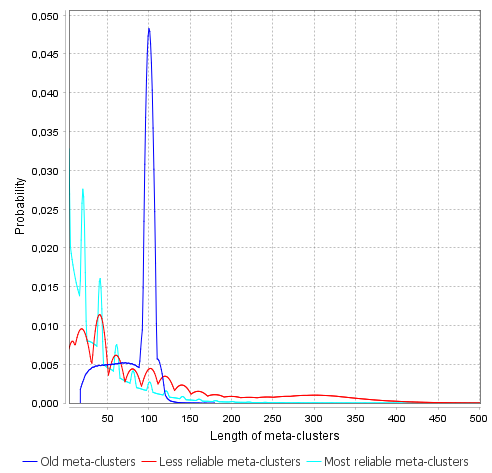


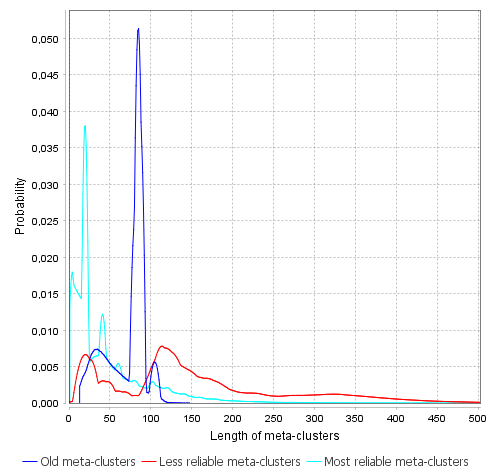


(b)


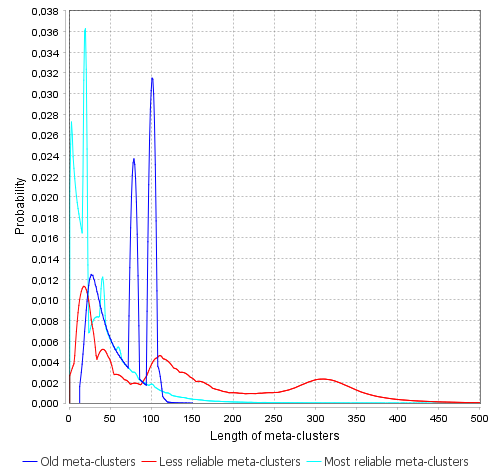


(c)

**Figure S3.** Densities of meta-clusters lengths for (a) ESR1, (b) JUN, and (c) FOXA1.

**Table S2.** AUC values for motif identification in meta-clusters of ESR1, JUN, and FOXA1, with the help of HOCOMOCO and TRANSFAC PWMs.

| **TF** | **Old meta-clusters** | **New less reliable meta-clusters** | **New most reliable meta-clusters** |
| --- | --- | --- | --- |
| ESR1  HOCOMOCO  TRANSFAC (V$ESR1_10) | 0.585  0.579 | 0.570  0.565 | 0.605  0.597 |
| JUN  HOCOMOCO  TRANSFAC (V$JUN_11) | 0.622  0.608 | 0.586  0.567 | 0.597  0.591 |
| FOXA1  HOCOMOCO  TRANSFAC ( V$FOXA1_14 ) | 0.689  0.653 | 0.587  0.598 | 0.699  0.658 |

a)
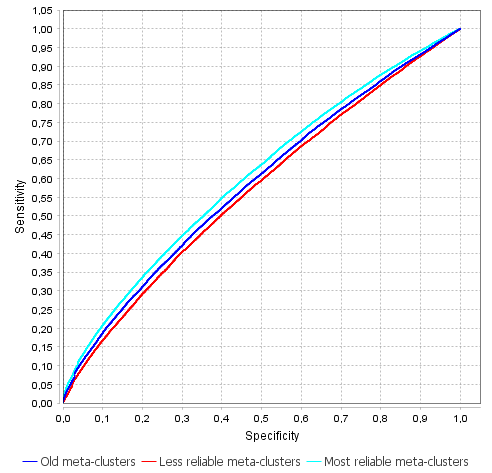


b)
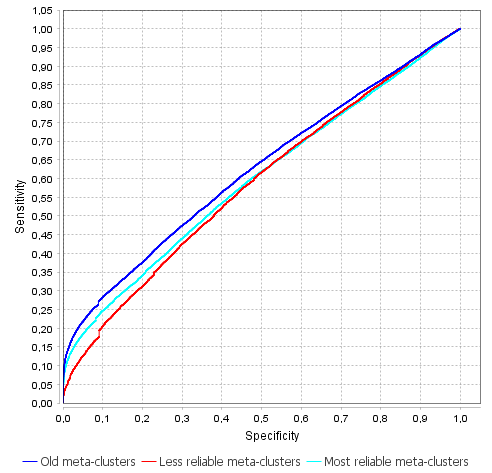


c)
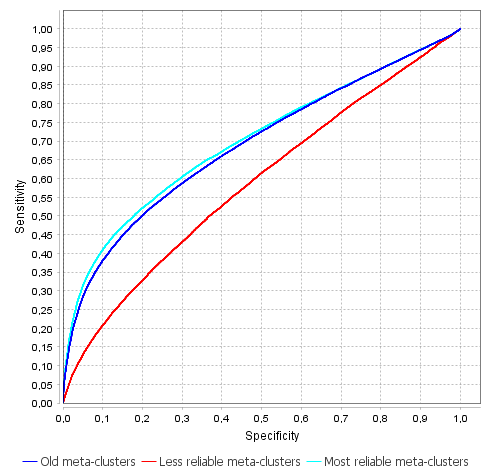


**Figure S4.** ROC curves for (a) ESR1, (b) JUN, and (c) FOXA1.

## REFERENCES FOR THE SUPPLEMENT 4

1. Kolmykov S. K., Kondrakhin Y. V., Yevshin I. S., Sharipov R. N., Ryabova A. S. and Kolpakov F. A. Population size estimation for quality control of ChIP-Seq datasets. *PLOS One* 2019, 14.

2. S. Lin S. Rank aggregation methods. *Wiley Interdisciplinary Reviews: Computational Statistics*, 2010 2: 555-570

3. Wang X., Li X, Xiao G. A comparative study of rank aggregation methods for partial and top ranked lists in genomic applications. *Briefings in bioinformatics*, 2019, 20:178-189.

4. Kolde R., Laur S., Adler P., Vilo J. Robust rank aggregation for gene list integration and meta-analysis, *Bioinformatics.* 2012, 28:573-580

5. Kel AE, Gossling E, Reuter I, Cheremushkin E, Kel-Margoulis OV, Wingender E. MATCHTM: a tool for searching transcription factor binding sites in DNA sequences. Nucleic Acids Res. 2003 Jul 1;31(13):3576–3579.

6. Mathelier, A. and Wasserman, W.W. (2013) The next generation of transcription factor binding site prediction, PLoS Comput Biol 9(9).

**Supplement 5**

**Master site visualization**

The master site is a further extension of the concept of the meta-cluster. A meta-cluster defines boundaries of the binding site (binding region, gray box in Fig. S5A). The master site for such a region integrates all information from the GTRD, including ChIP-seq peaks (red boxes), motif hits from sequence scanning with position weight matrices (pink boxes), overlapping open chromatin regions (dark green regions), DNase footprints (light green boxes) and histone modification marks (yellow boxes). BioUML genome browser allows to show/hide various details about the master site (Fig. S5B).


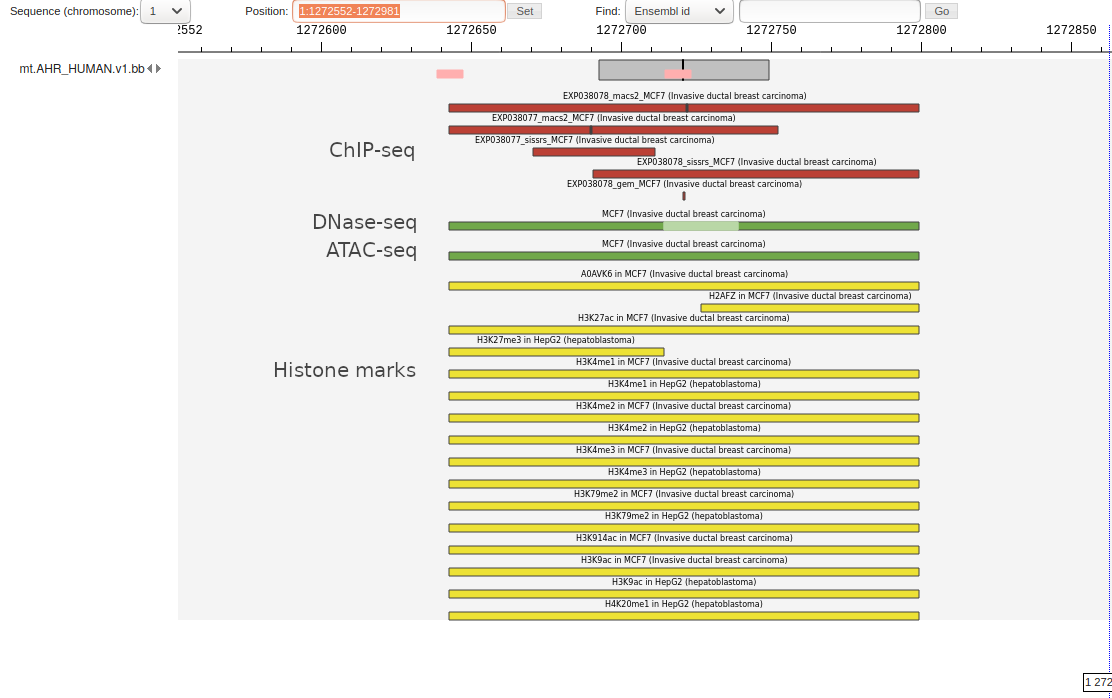


**Figure S5A.** Visualization of master site content by the BioUML genome browser.


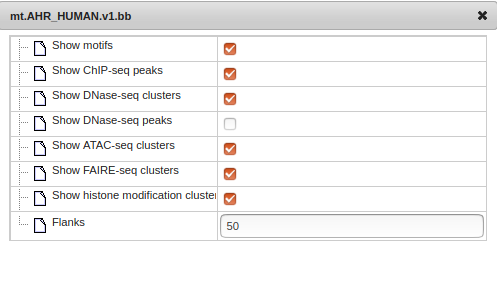


**Figure S5B.** Master site visualization options.

**Supplement 6**

**Track finder**

The GTRD contains a huge number of tracks (Supplement 8). To allow users to find tracks of interest by different criteria and visualize them in a genome browser, we have developed a special pane – Track finder. Figures S6 and S7 demonstrate its interface. First, a user can choose the NGS data type. Then, the user can select the cell type using hierarchical classification (clusters). Finally, the user can specify the TF of interest. When all parameters are chosen, a user needs to push the “Start” button (highlighted by a red square). All search results will be presented in the right panel. Also, all search results can be shown in the genome browser by using the “Show” button.


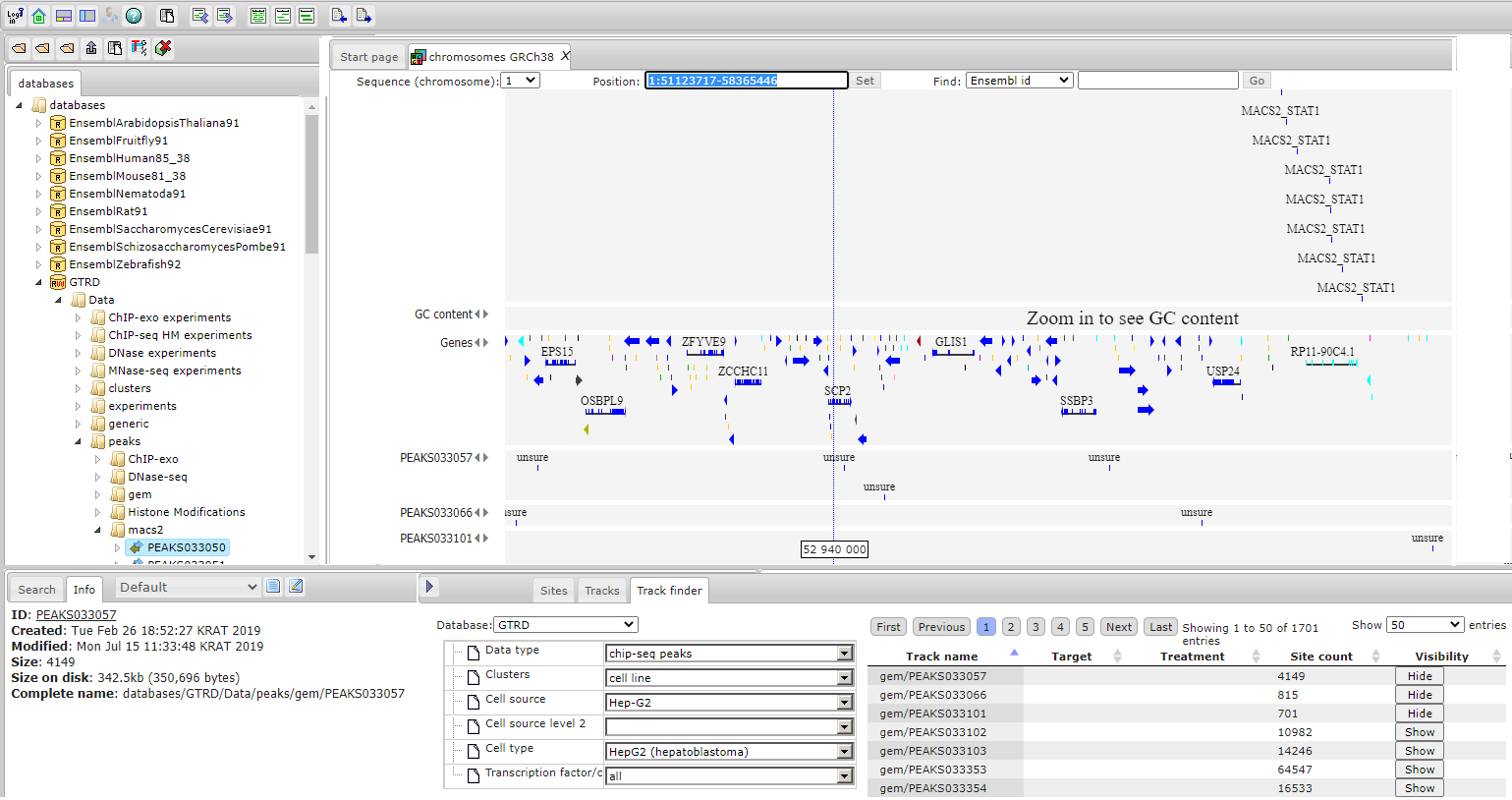


**Figure S6**. Track finder panel as part of the BioUML genome browser.


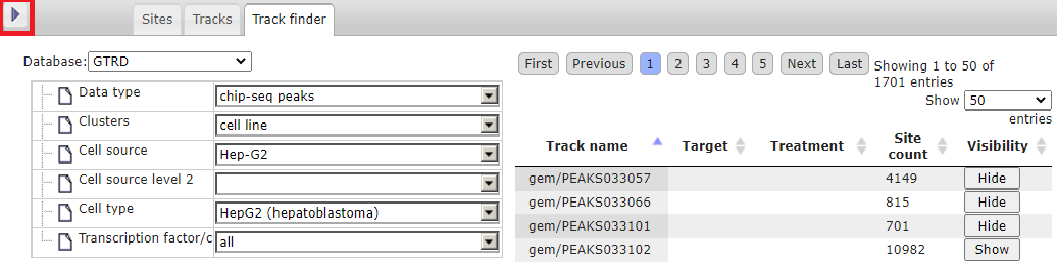


**Figure S7**. Track finder interface.

**Supplement 7**

**Statistics on different experiment types annotated in the GTRD**

|  | **Transcription factors and cofactors** | | **Histone modifica-tions,**  **ChIP-seq** | **Open chromatin** | | | | **Allele**  **specific**  **binding** |
| --- | --- | --- | --- | --- | --- | --- | --- | --- |
|  | ChIP-seq | ChIP-exo |  | DNase-  seq | ATAC-  seq | MNase-  seq | FAIRE-  seq |  |
| *Mus musculus* | 12 519 | 88 | 1442 | 190 | 5 695 | 352 | 131 |  |
| *Homo sapiens* | 15 982 | 362 | 4349 | 1 669 | 4 039 | 154 | 237 | for 1033  TFs  in 579 cell types |
| *Rattus norvegicus* | 279 | 2 | 67 | 12 | 29 | 4 |  |  |
| *Drosophila melanogaster* | 2 571 | 47 | 72 | 14 | 270 | 214 | 58 |  |
| *Caenorhabditis elegans* | 1 642 |  | 12 | 94 | 46 | 31 |  |  |
| *Arabidopsis thaliana* | 493 |  | 45 |  | 38 | 53 | 8 |  |
| *Saccharomyces cerevisiae* | 1 765 | 305 | 126 | 9 | 112 | 599 | 102 |  |
| *Danio rerio* | 84 |  |  |  | 159 | 6 | 4 |  |
| *Schizosaccharomyces pombe* | 384 | 13 | 30 |  |  | 92 |  |  |
| GTRD version 20.06 | 35 719 | 821 | 6 153 | 1 988 | 10 442 | 1 507 | 540 |  |
| GTRD version 19.04  public release | 24 634 | - | - | 1 585 | - | - | - |  |

**Supplement 8**

**GTRD tracks statistics**

| **Data type** | **Alignments** | **Tracks** | | |
| --- | --- | --- | --- | --- |
|  |  | **Type** | **Count** | **Total** |
| ChIP-seq | 35 669  (control  11 082) | MACS2  GEM  SISSRS  PICS  Combined by TF tracks (MACS2, GEM, SISSRS, PICS)  Clusters by TF  Meta-clusters by TF  Combined (union) tracks (MACS2, GEM, SISSRS, PICS)  Clusters  Meta-clusters | 23 425  24 242  24 025  * 24 322  14 116  14 116  3 529  36 (4x9)  36 (4x9)  9 | 127 676 |
| DNase-seq | 2 284 | MACS2  Wellington (MACS2)  Hotspot2  Wellington (Hotspot2) | 1 906  1 894  1 905  1 904 | 7 609 |
| ChIP-exo | 807  (control 45) | GEM  Peakzilla | 680  603 | 1 283 |
| ChIP-seq HM | 14 145  (control 2 322) | MACS2 | 11 823 | 11 823 |
| MNase-seq | 1 071 | Danpos2 | 1 056 | 2 127 |
| ATAC-seq | 10 520 | MACS2 | 10 520 | 10 520 |
| FAIRE-seq | 442 | MACS2 | 442 | 442 |
| **Total** | **64 938** |  |  | **161 480** |

* While the initial number of alignments processed by different peak callers is the same, they have produced different track numbers due to the low quality of some data.
